# Activation of the Calcium Sensing Receptor Decreases Secretagogue-Induced Fluid Secretion in the Rat Small Intestine

**DOI:** 10.3389/fphys.2019.00439

**Published:** 2019-05-03

**Authors:** Maria J. Barahona, Renee M. Maina, Taras Lysyy, Michele Finotti, Giorgio Caturegli, Vanessa Baratta, Francesco D’Amico, David Mulligan, John P. Geibel

**Affiliations:** ^1^Department of Surgery, Yale School of Medicine, New Haven, CT, United States; ^2^Transplantation and Hepatobiliary Surgery, University of Padua, Padua, Italy

**Keywords:** CaSR, FITC-inulin, fluid homeostasis, forskolin, gastrointestinal tract

## Abstract

**Background:**

The calcium-sensing receptor (CaSR) has been localized and characterized in numerous tissues throughout the body. In the mammalian gastrointestinal tract, the CaSR is known to act as a nutrient sensor and has recently been found to play a role in intestinal fluid and electrolyte balance. This study aims to demonstrate the functionality of the CaSR as a modulator of fluid secretion and absorption along the small intestine.

**Methods:**

Small intestine regions (proximal, middle, and distal) were isolated from Sprague Dawley rats and loaded into an *ex vivo* intestinal perfusion device that provides independent intraluminal and extraluminal (serosa/basolateral) perfusion. The regions were perfused with 5 and 7 mM of Ca^2+^, both in the presence and absence of forskolin (FSK), a potent secretagogue. Control experiments were conducted with intraluminal perfusate containing standard Ringer-HEPES buffer with a physiological concentration of Ca^2+^ (1 mM). A second set of comparison experiments was performed with intraluminal perfusates containing AC-265347, a CaSR activator and agonist, in the presence of FSK. In all experimental conditions, the intraluminal perfusate contained fluorescein isothiocyanate (FITC)-inulin, a nonabsorbable fluorescent marker of secretion and/or absorption. Intraluminal fluorescence signal was utilized as a measure of water movement at the start of the experiment and every 15 min for 90 min.

**Results:**

Under physiological conditions, increasing the concentration of Ca^2+^ in the luminal perfusate reduced intestinal fluid secretion in all regions. At a Ca^2+^ concentration of 7 mM, net fluid absorption was observed in all regions. In the presence of FSK, 5 mM Ca^2+^ significantly decreased fluid secretion and 7 mM Ca^2+^ abolished FSK-induced fluid secretion. Intraluminal perfusion with 5 mM Ca^2+^ was as effective as AC-265347, in reducing secretagogue-induced fluid hypersecretion in the proximal and middle regions.

**Conclusion:**

This study concludes that apical CaSR is active along the small intestine. Its activation by Ca^2+^ and/or calcimimetics reduces fluid secretion in a dose-dependent manner, with higher Ca^2+^ concentrations, or application of a calcimimetic, leading to fluid absorption. We furthermore show that, in the presence of FSK, receptor activation abates FSK secretagogue-induced fluid secretion. This presents a new therapeutic target to address secretory diarrheal illnesses.

## Introduction

The extracellular CaSR is a classic transmembrane GPCR that was first identified in the parathyroid gland, where it plays an important role in total body calcium (Ca^2+^) homeostasis (Brown et al., 1993; Brown and MacLeod, 2001). In the mammalian gastrointestinal tract, the CaSR has been identified in Meissner’s and Auerbach’s plexuses as well as the apical and basolateral membranes of fluid-absorbing villous cells and fluid-secreting crypt cells of rat and human intestines (Chattopadhyay et al., 1998; Sheinin et al., 2000; Cheng et al., 2002; Tfelt-Hansen and Brown, 2005; Cheng, 2012; Macleod, 2013). In the absorptive gut, the CaSR functions as a nutrient sensor and provides a mechanism to signal to the enteric nervous system and coordinate food delivery and gut motility in order to maximize nutrient absorption (Tang et al., 2016). It has also been implicated in intestinal ion transport and the regulation of bicarbonate secretion (Xie et al., 2014; Tang et al., 2015).

In rat colonic crypts, the CaSR has been shown to be activated by Ca^2+^, amino acids, polyamines, and calcimimetics, resulting in net inhibition of fluid secretion into the lumen (Cheng et al., 2004; Geibel et al., 2006). The receptor modulates fluid and electrolyte secretion in the colon, both under normal physiological conditions and following exposure to potent secretagogues such as FSK, cholera toxin and heat-stable enterotoxins (Flores and Sharp, 1976; Geibel et al., 2006; Breitwieser, 2013; Tang et al., 2015). Extensive studies have been done on the role of the CaSR in the colon, which is not the case in the small intestine. Although the CaSR has been found along the entire length of the small intestine (Cheng et al., 2002; Dufner et al., 2005; Tu and Bikle, 2013; Alfadda et al., 2014), functional data on its role in modulating salt and fluid transport in the small intestine is lacking, partly due to the difficulty of isolating and maintaining viable villi. In this study, an *ex vivo* perfusion device capable of providing real-time data on intestinal fluid absorption and secretion is used to examine the functional role of the CaSR on the apical surface along the length of the small intestine. We also study the functional role of activation of the CaSR in blocking cyclic nucleotide-induced intestinal fluid secretion.

## Materials and Methods

Male Sprague-Dawley rats weighing between 250 and 490 g (*n* = 24) were acquired from Charles River Laboratories (Wilmington, MA, United States) and housed in climate-controlled and light-cycled rooms with free access to water, in accordance with Yale University Animal Care and Use Committee guidelines (Protocol #2018-10253). The rats were fasted 12–15 h prior to each experiment.

### Small Intestine Procurement

Fasted rats were fully anesthetized with Isoflurane (IsoThesia^TM^, 99.9%/mL) after which a midline laparotomy was performed. Cold (4°C) Ringer-HEPES buffer (Table 1) was used to irrigate the abdominal cavity to reduce endogenous metabolism and decrease ischemic damage. The entire small bowel was procured. The proximal region (jejunum) was recovered 10–12 cm distally from the stomach and the distal region (ileum) was obtained 1–2 cm proximally from the ileocecal junction. The midpoint of the residual intestinal region was identified, and the middle region was obtained by cutting off 5 cm above and 5 cm below the midpoint. Each intestinal region (proximal, middle, and distal) was approximately 10 cm in length. The lumen of the regions was flushed with cold Ringer-HEPES buffer to remove residual fecal material. The regions were then connected to perfusion chambers of an *ex vivo* perfusion device previously described by our group (Munoz-Abraham et al., 2015). Each chamber provided independent serosa (extraluminal) and luminal (intraluminal) perfusion to the intestinal regions at a constant temperature and flow rate (37°C and 6 mL/min).

**Table 1:** Perfusion solutions.

Solution	Composition
Standard Ringer-HEPES buffer	117 mM NaCl, 5 mM KCl, 1 mM CaCl_2_^∗^2H_2_O, 1.2 mM MgSO_4_^∗^7 H_2_O, 32.2 mM HEPES, 10 mM Glucose
Cold Ringer-HEPES buffer	101 mM NaCl, 5 mM KCl, 1 mM CaCl_2_^∗^2 H_2_O, 1.2 mM MgSO_4_^∗^7 H_2_O, 32.2 mM HEPES, 10 mM Glucose
5 mM Ca^2+^	109 mM NaCl, 5 mM KCl, 5 mM CaCl_2_^∗^2H_2_O, 1.2 mM MgSO_4_^∗^7 H_2_O, 32.2 mM HEPES, 10 mM Glucose
7 mM Ca^2+^	105 mM NaCl, 5 mM KCl, 7 mM CaCl_2_^∗^2H_2_O, 1.2 mM MgSO_4_^∗^7 H_2_O, 32.2 mM HEPES, 10 mM Glucose

### *Ex vivo* Perfusion

The first set of experiments included two groups in which the intestinal regions were intraluminally perfused with Ringer-HEPES buffer solution containing different concentrations of Ca^2+^ (5 and 7 mM) separately. Control regions were perfused with standard Ringer-HEPES buffer containing 1 mM Ca^2+^, which is within the physiologic concentration of Ca^2+^ in the small intestine (Chattopadhyay, 2000). The second set of experiments included three groups, in which the intraluminal perfusate contained 10 μM FSK (Sigma-Aldrich, St. Louis, MO, United States) a potent secretagogue, and varying concentrations of Ca^2+^ (5, 7, and control 1 mM), respectively. Lastly, we compared the effect of 1, 5, and 7 mM intraluminal Ca^2+^ in the presence of FSK to that of 2 μM of the calcimimetic 1-Benzothiazol-2-yl-1-(2,4-dimethyl-phenyl)-ethanol (AC-265347, Sigma-Aldrich, St. Louis, MO, United States), which is an allosteric agonist of the CaSR, which is also known to reduce ionized calcium (Ma et al., 2011).

In summary, a total of seven experimental conditions were performed for each intestinal region. In each condition extraluminal perfusion was with Ringer-HEPES buffer and the intraluminal perfusates were as follows:

(1) Ringer-HEPES buffer with 5 mM Ca^2+^
(2) Ringer-HEPES buffer with 5 mM Ca^2+^ + 10 μM FSK
(3) Ringer-HEPES buffer with 7 mM Ca^2+^
(4) Ringer-HEPES buffer with 7 mM Ca^2+^ + 10 μM FSK
(5) Standard Ringer-HEPES buffer
(6) Standard Ringer-HEPES buffer + 10 μM FSK
(7) Standard Ringer-HEPES buffer + 10 μM FSK + 2 μM AC-265347

All intraluminal perfusates also contained 1 mM FITC-inulin, a non-absorbable fluorescent marker of fluid secretion and/or absorption (Munoz-Abraham et al., 2015; AlKukhun et al., 2017). All solutions were adjusted to a final osmolarity of 300 ± 5 mOsm with mannitol, at a pH of 7.4 and temperature of 37°C. The concentration of NaCl was appropriately adjusted to compensate for the increased concentration of CaCl_2_, thereby keeping chloride concentration constant (refer to Table 1 for composition of all perfusion solutions).

### Measurement of Fluid Secretion and Absorption

Nanofluorospectrophotometry (Nanodrop 3300, Thermo Fisher Scientific Inc.) was used to measure fluorescence intensity of the intraluminal perfusate over time. The greater the fluorescence of the sampled luminal content, the less water secretion into the lumen was observed, decreases in luminal fluorescence were indicative of a decreased concentration of FITC-inulin. For each experiment, fluorescence measurements were taken at seven-time points over the course of the experiment: 0, 15, 30, 45, 60, 75, and 90 min. Five intraluminal fluid samples of 2 μL each were taken for each experimental time point; the slope and SEM were calculated and graphed. At the end of each experiment, extraluminal perfusate samples were collected to exclude the possibility of leakage due to perforation or damage to the intestinal wall. The absence of any detected FITC-inulin in the basolateral (extraluminal) perfusate ruled out leakage and confirmed preserved integrity of intestinal regions. Presence of FITC-inulin in the extraluminal perfusate warranted discarding of the intestinal region.

### Data Presentation and Statistical Analysis

Intraluminal fluorescence values of FITC-inulin concentration were calculated, plotted and analyzed using GraphPad Prism 8.01 (GraphPad Software). Linear regression analysis was performed to determine slopes, which represented average secretion or absorption per minute over 90 min. Statistically significant differences were calculated using mixed-effects analysis and Tukey’s multiple comparisons test. A *p* value of less than 0.05 (*p* < 0.05) was considered statically significant.

## Results

### Effect of Increasing Ca^2+^ Concentration

**FIGURE 1:**
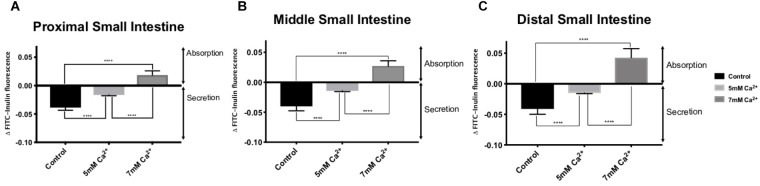
Effect of increasing intraluminal [Ca^2+^] on basal intestinal fluid secretion in the: **(A)** Proximal small intestine **(B)** Middle small intestine **(C)** Distal small intestine. *P* value < 0.0001 is represented by ^∗∗∗∗^.

In the absence of FSK in the intraluminal perfusate, all small intestinal regions exhibited basal secretion with standard Ringer-HEPES buffer containing 1 mM Ca^2+^ (Table 2). When the intraluminal Ca^2+^ concentration was increased to 5 mM, the average secretion over time significantly decreased in all intestinal regions (Figure 1). Intraluminal perfusion with 7 mM Ca^2+^ not only abolished fluid secretion but also caused moderate fluid absorption in all intestinal regions, reversing the typical endogenous secretion profile seen in control regions perfused with standard Ringer-HEPES (Figure 1).

**Table 2:** Δ Fluorescence/Minute Mean with varying [Ca^2+^].

	Proximal	Middle	Distal	N^∗^
Control (1 mM Ca^2+^)	−0.039	−0.040	−0.041	3
5 mM Ca^2+^	−0.016	−0.014	−0.015	3
7 mM Ca^2+^	0.019	0.027	0.043	3

*Negative values represent the mean of fluid secretion while positive values represent the mean of fluid absorption. As the intraluminal Ca^2+^ increases, the rate of basal fluid secretion exhibited by the intestinal regions decreases. Fluid absorption is seen at a Ca^2+^ concentration of 7 mM. N^∗^ is the number of animals used in each group.*

### Presence of FSK

**FIGURE 2:**
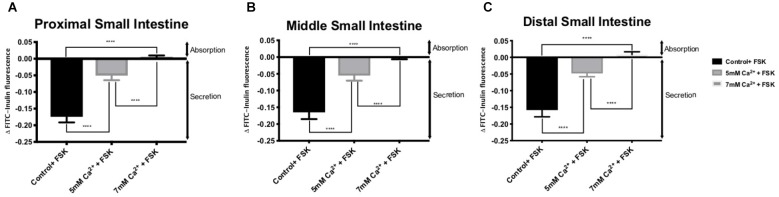
Intestinal fluid secretion in the presence of intraluminal 10 μM forskolin with varying [Ca^2+^] in the: **(A)** Proximal small intestine **(B)** Middle small intestine **(C)** Distal small intestine. *P* value < 0.0001 is represented by ^∗∗∗∗^.

Addition of FSK to the intraluminal perfusate dramatically increased fluid secretion in the control intestinal regions perfused with standard Ringer-HEPES (Table 3). In the presence of 5 mM Ca^2+^, intraluminal fluid secretion in all regions perfused with FSK significantly decreased in comparison to the controls (*p* < 0.0001 in all regions). As shown in Figure 2, intraluminal perfusion with 7 mM Ca^2+^ completely abolished FSK-induced secretion in all intestinal regions. This effect was statistically significant in comparison to control experiments (*p* < 0.0001 in all regions).

**Table 3:** ΔFluorescence/Minute Mean in the presence of intraluminal FSK.

	Proximal	Middle	Distal	N^∗^
Control (1 mM Ca^2+^)	−0.175	−0.165	−0.158	3
5 mM Ca^2+^	−0.051	−0.006	−0.048	3
7 mM Ca^2+^	−0.004	−0.001	0.008	3
1 mM Ca^2+^ + AC 256437	−0.069	−0.08273	−0.04758	3

*Increasing intraluminal Ca^2+^ decreases the mean fluid secretion induced by FSK. N^∗^ is the number of animals used in each group.*

**Table 4:** Proximal region of small intestine. *P* values of ΔFluorescence/Minute with the different [Ca^2+^] and FSK in comparison to AC-265347 with FSK.

Experimental group	Experimental group	*P* value
1 mM Ca^2+^ HEPES + FITC	1 mM Ca^2+^ HEPES + FITC + 10 μM FSK	<0.0001
1 mM Ca^2+^ HEPES + FITC	5 mM Ca^2+^ HEPES + FITC	<0.0001
1 mM Ca^2+^ HEPES + FITC	5 mM Ca^2+^ HEPES + FITC + 10 μM FSK	<0.0001
1 mM Ca^2+^HEPES + FITC	7 mM Ca^2+^ HEPES + FITC	<0.0001
1 mM Ca^2+^ HEPES + FITC	7 mM Ca^2+^ HEPES + FITC + 10 μM FSK	<0.0001
1 mM Ca^2+^ HEPES + FITC	1 mM Ca^2+^ + 10 μM FSK + 2 μM AC 256437	**0.6803 (ns)**
1 mM Ca^2+^ HEPES + FITC + 10 μM FSK	5 mM Ca^2+^ HEPES + FITC	<0.0001
1 mM Ca^2+^ HEPES + FITC + 10 μM FSK	5 mM Ca^2+^ HEPES + FITC + 10 μM FSK	<0.0001
1 mM Ca^2+^ HEPES + FITC + 10 μM FSK	7 mM Ca^2+^ HEPES + FITC	<0.0001
1 mM Ca^2+^ HEPES + FITC + 10 μM FSK	7 mM Ca^2+^ HEPES + FITC + 10 μM FSK	<0.0001
1 mM Ca^2+^ HEPES + FITC + 10 μM FSK	1 mM Ca^2+^ + 10 μM FSK + 2 μM AC 256437	<0.0001
5 mM Ca^2+^ HEPES + FITC	5 mM Ca^2+^ HEPES + FITC + 10 μM FSK	<0.0001
5 mM Ca^2+^ HEPES + FITC	7 mM Ca^2+^ HEPES + FITC	<0.0001
5 mM Ca^2+^ HEPES + FITC	7 mM Ca^2+^ HEPES + FITC + 10 μM FSK	**0.5387 (ns)**
5 mM Ca^2+^ HEPES + FITC	1 mM Ca^2+^ + 10 μM FSK + 2 μM AC 256437	**0.0019**
5 mM Ca^2+^ HEPES + FITC + 10 μM FSK	7 mM Ca2+ HEPES + FITC	<0.0001
5 mM Ca^2+^ HEPES + FITC + 10 μM FSK	7 mM Ca^2+^ HEPES + FITC + 10 μM FSK	<0.0001
5 mM Ca^2+^ HEPES + FITC + 10 μM FSK	1 mM Ca^2+^ + 10 μM FSK + 2 μM AC 256437	**0.6634 (ns)**
7 mM Ca^2+^ HEPES + FITC	7 mM Ca^2+^ HEPES + FITC + 10 μM FSK	<0.0001
7 mM Ca^2+^ HEPES + FITC	1 mM Ca^2+^ + 10 μM FSK + 2 μM AC 256437	<0.0001
7 mM Ca^2+^ HEPES + FITC + 10 μM FSK	1 mM Ca^2+^ + 10 μM FSK + 2 μM AC 256437	0.0004
**Experimental group**	Experimental group	*P* value
1 mM Ca^2+^ HEPES + FITC	1 mM Ca^2+^ HEPES + FITC + 10 μM FSK	<0.0001
1 mM Ca^2+^ HEPES + FITC	5 mM Ca^2+^ HEPES + FITC	<0.0001
1 mM Ca^2+^ HEPES + FITC	5 mM Ca^2+^ HEPES + FITC + 10 μM FSK	<0.0001
1 mM Ca^2+^HEPES + FITC	7 mM Ca^2+^ HEPES + FITC	<0.0001
1 mM Ca^2+^ HEPES + FITC	7 mM Ca^2+^ HEPES + FITC + 10 μM FSK	<0.0001
1 mM Ca^2+^ HEPES + FITC	1 mM Ca^2+^ + 10 μM FSK + 2 μM AC 256437	**0.6803 (ns)**
1 mM Ca^2+^ HEPES + FITC + 10 μM FSK	5 mM Ca^2+^ HEPES + FITC	<0.0001
1 mM Ca^2+^ HEPES + FITC + 10 μM FSK	5 mM Ca^2+^ HEPES + FITC + 10 μM FSK	<0.0001
1 mM Ca^2+^ HEPES + FITC + 10 μM FSK	7 mM Ca^2+^ HEPES + FITC	<0.0001
1 mM Ca^2+^ HEPES + FITC + 10 μM FSK	7 mM Ca^2+^ HEPES + FITC + 10 μM FSK	<0.0001
1 mM Ca^2+^ HEPES + FITC + 10 μM FSK	1 mM Ca^2+^ + 10 μM FSK + 2 μM AC 256437	<0.0001
5 mM Ca^2+^ HEPES + FITC	5 mM Ca^2+^ HEPES + FITC + 10 μM FSK	<0.0001
5 mM Ca^2+^ HEPES + FITC	7 mM Ca^2+^ HEPES + FITC	<0.0001
5 mM Ca^2+^ HEPES + FITC	7 mM Ca^2+^ HEPES + FITC+10 μM FSK	**0.5387 (ns)**
5 mM Ca^2+^ HEPES + FITC	1 mM Ca^2+^ + 10 μM FSK + 2 μM AC 256437	**0.0019**
5 mM Ca^2+^ HEPES + FITC + 10 μM FSK	7 mM Ca2+ HEPES + FITC	<0.0001
5 mM Ca^2+^ HEPES + FITC + 10 μM FSK	7 mM Ca^2+^ HEPES + FITC + 10 μM FSK	<0.0001
5 mM Ca^2+^ HEPES + FITC + 10 μM FSK	1 mM Ca^2+^ + 10 μM FSK + 2 μM AC 256437	**0.6634 (ns)**
7 mM Ca^2+^ HEPES + FITC	7 mM Ca^2+^ HEPES + FITC + 10 μM FSK	<0.0001
7 mM Ca^2+^ HEPES + FITC	1 mM Ca^2+^ + 10 μM FSK + 2 μM AC 256437	<0.0001
7 mM Ca^2+^ HEPES + FITC + 10 μM FSK	1 mM Ca^2+^ + 10 μM FSK + 2 μM AC 256437	0.0004

*Proximal small intestine segments/sections p value obtained with Tukey’s multiple comparisons test. A p value <0.05 was considered statistically significant. ns means non-significant.*

**Table 5:** Middle region of small intestine. *P* values of ΔFluorescence/Minute with the different [Ca^2+^] and FSK in comparison to AC-265347 with FSK.

Experimental group	Experimental group	*P* value
1 mM Ca^2+^ HEPES + FITC	1 mM Ca^2+^ HEPES + FITC + 10 μM FSK	<0.0001
1 mM Ca^2+^ HEPES + FITC	5 mM Ca^2+^ HEPES + FITC	<0.0001
1 mM Ca^2+^ HEPES + FITC	5 mM Ca^2+^ HEPES + FITC + 10 μM FSK	**0.9997 (ns)**
1 mM Ca^2+^HEPES + FITC	7 mM Ca^2+^ HEPES + FITC	<0.0001
1 mM Ca^2+^ HEPES + FITC	7 mM Ca^2+^ HEPES + FITC + 10 μM FSK	<0.0001
1 mM Ca^2+^ HEPES + FITC	1 mM Ca^2+^ + 10 μM FSK + 2 μM AC 256437	**0.9562 (ns)**
1 mM Ca^2+^ HEPES + FITC + 10 μM FSK	5 mM Ca^2+^ HEPES + FITC	<0.0001
1 mM Ca^2+^ HEPES + FITC + 10 μM FSK	5 mM Ca^2+^ HEPES + FITC + 10 μM FSK	<0.0001
1 mM Ca^2+^ HEPES + FITC + 10 μM FSK	7 mM Ca^2+^ HEPES + FITC	<0.0001
1 mM Ca^2+^ HEPES + FITC + 10 μM FSK	7 mM Ca^2+^ HEPES + FITC + 10 μM FSK	<0.0001
1 mM Ca^2+^ HEPES + FITC + 10 μM FSK	1 mM Ca^2+^ + 10 μM FSK + 2 μM AC 256437	<0.0001
5 mM Ca^2+^ HEPES + FITC	5 mM Ca^2+^ HEPES + FITC + 10 μM FSK	<0.0001
5 mM Ca^2+^ HEPES + FITC	7 mM Ca^2+^ HEPES + FITC	<0.0001
5 mM Ca^2+^ HEPES + FITC	7 mM Ca^2+^ HEPES + FITC+10 μM FSK	**0.0016**
5 mM Ca^2+^ HEPES + FITC	1 mM Ca^2+^ + 10 μM FSK + 2 μM AC 256437	<0.0001
5 mM Ca^2+^ HEPES + FITC + 10 μM FSK	7 mM Ca^2+^ HEPES + FITC	<0.0001
5 mM Ca^2+^ HEPES + FITC + 10 μM FSK	7 mM Ca^2+^ HEPES + FITC + 10 μM FSK	<0.0001
5 mM Ca^2+^ HEPES + FITC + 10 μM FSK	1 mM Ca^2+^ + 10 μM FSK + 2 μM AC 256437	**0.8971 (ns)**
7 mM Ca^2+^ HEPES + FITC	7 mM Ca^2+^ HEPES + FITC + 10 μM FSK	<0.0001
7 mM Ca^2+^ HEPES + FITC	1 mM Ca^2+^ + 10 μM FSK + 2 μM AC 256437	<0.0001
7 mM Ca^2+^ HEPES + FITC + 10 μM FSK	1 mM Ca^2+^ + 10 μM FSK + 2 μM AC 256437	<0.0001

*Middle small intestine segments/sections p value obtained with Tukey’s multiple comparisons test. A p value <0.05 was considered statistically significant. ns means non-significant.*

**Table 6:** Distal region of small intestine. *P* values of ΔFluorescence/Minute with the different [Ca^2+^] and FSK in comparison to AC-265347 with FSK.

Experimental group	Experimental group	*P* value
1 mM Ca^2+^ HEPES + FITC	1 mM Ca^2+^ HEPES + FITC + 10 μM FSK	<0.0001
1 mM Ca^2+^ HEPES + FITC	5 mM Ca^2+^ HEPES + FITC	<0.0001
1 mM Ca^2+^ HEPES + FITC	5 mM Ca^2+^ HEPES + FITC + 10 μM FSK	**0.6364 (ns)**
1 mM Ca^2+^HEPES + FITC	7 mM Ca^2+^ HEPES + FITC	<0.0001
1 mM Ca^2+^ HEPES + FITC	7 mM Ca^2+^ HEPES + FITC + 10 μM FSK	<0.0001
1 mM Ca^2+^ HEPES + FITC	1 mM Ca^2+^ + 10 μM FSK + 2 μM AC 256437	**0.0628 (ns)**
1 mM Ca^2+^ HEPES + FITC + 10 μM FSK	5 mM Ca^2+^ HEPES + FITC	<0.0001
1 mM Ca^2+^ HEPES + FITC + 10 μM FSK	5 mM Ca^2+^ HEPES + FITC + 10 μM FSK	<0.0001
1 mM Ca^2+^ HEPES + FITC + 10 μM FSK	7 mM Ca^2+^ HEPES + FITC	<0.0001
1 mM Ca^2+^ HEPES + FITC + 10 μM FSK	7 mM Ca^2+^ HEPES + FITC + 10 μM FSK	<0.0001
1 mM Ca^2+^ HEPES + FITC + 10 μM FSK	1 mM Ca^2+^ + 10 μM FSK + 2 μM AC 256437	<0.0001
5 mM Ca^2+^ HEPES + FITC	5 mM Ca^2+^ HEPES + FITC + 10 μM FSK	<0.0001
5 mM Ca^2+^ HEPES + FITC	7 mM Ca^2+^ HEPES + FITC	<0.0001
5 mM Ca^2+^ HEPES + FITC	7 mM Ca^2+^ HEPES + FITC+10 μM FSK	**0.0442**
5 mM Ca^2+^ HEPES + FITC	1 mM Ca^2+^ + 10 μM FSK + 2 μM AC 256437	<0.0001
5 mM Ca^2+^ HEPES + FITC + 10 μM FSK	7 mM Ca^2+^ HEPES + FITC	<0.0001
5 mM Ca^2+^ HEPES + FITC + 10 μM FSK	7 mM Ca^2+^ HEPES + FITC + 10 μM FSK	<0.0001
5 mM Ca^2+^ HEPES + FITC + 10 μM FSK	1 mM Ca^2+^ + 10 μM FSK + 2 μM AC 256437	**0.0034**
7 mM Ca^2+^ HEPES + FITC	7 mM Ca^2+^ HEPES + FITC + 10 μM FSK	<0.0001
7 mM Ca^2+^ HEPES + FITC	1 mM Ca^2+^ + 10 μM FSK + 2 μM AC 256437	<0.0001
7 mM Ca^2+^ HEPES + FITC + 10 μM FSK	1 mM Ca^2+^ + 10 μM FSK + 2 μM AC 256437	<0.0001

*Distal small intestine segments/sections p value obtained with Tukey’s multiple comparisons test. A p value <0.05 was considered statistically significant. ns means non-significant.*

### Comparison With Calcimimetic AC-265347

**FIGURE 3:**
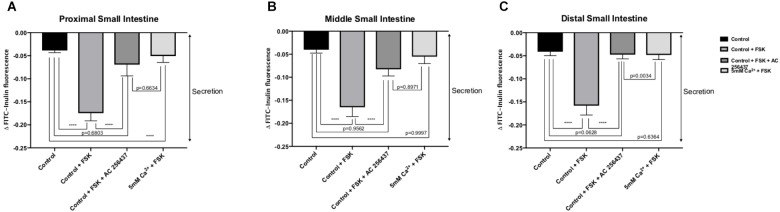
Comparison of the effect of intraluminal 1 mM [Ca^2+^] as control, 5 mM [Ca^2+^] and 2 μM AC-265347 with 1 mM [Ca^2+^] in reducing FSK-induced fluid secretion in the: **(A)** Proximal small intestine **(B)** Middle small intestine **(C)** Distal small intestine. *P* value < 0.0001 is represented by ^∗∗∗∗^.

**FIGURE 4:**
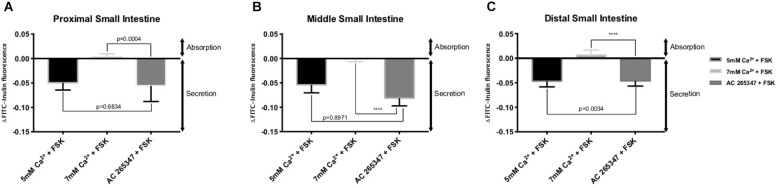
Comparison of the effect of intraluminal [Ca^2+^] and 2 μM AC-265347 in reducing FSK-induced fluid secretion in the: **(A)** Proximal small intestine **(B)** Middle small intestine **(C)** Distal small intestine. *P* value < 0.0001 is represented by ^∗∗∗∗^.

In order to determine if the CaSR was being activated with Ca^2+^, the secretion profiles in all the intestinal regions were compared in the presence of control (1 mM of Ca^2+^), control with 10 μM FSK, control with 10 μM FSK and 2 μM AC-265347, and 5 mM Ca^2+^ with 10 μM FSK. There was no statistical significance between control and control with 10 μM FSK and 2 μM AC-265347, a CaSR agonist, in all regions as shown in Figure 3. The comparisons of the effects of 5 and 7 mM Ca^2+^ in abating secretagogue-induced fluid secretion to that of 2 μM AC-265347 in standard Ringer-HEPES buffer containing 1 mM Ca^2+^ demonstrated that intraluminal perfusion with 5 mM Ca^2+^ in the presence of 10 μM FSK was statistically similar to intraluminal perfusion with 2 μM AC-265347 with 1 mM Ca^2+^ in decreasing FSK-induced fluid secretion in the proximal and middle regions of the intestine (*p* = 0.6634, *p* = 0.8971, respectively), and more effective in the distal region (*p* = 0.0034). The reduction in secretion observed with 7 mM Ca^2+^ was larger when compared to the calcimimetic AC-265347 with 1 mM of Ca^2+^ in the middle and distal regions (*p* < 0.0001) and had a similar effect in the proximal region (*p* = 0.0004) as observed in Figure 4. The mean and SEM from each condition were graphed and Tukey’s multiple comparisons test provided the adjusted *p* value for the intestinal regions’ comparison. A detail of each comparison is shown in Tables 4–6.

## Discussion

This study provides evidence that activation of the CaSR by Ca^2+^ or a calcimimetic decreases intraluminal fluid secretion under physiological conditions. Increasing luminal calcium concentrations completely abates the secretory effect of the potent secretagogue FSK along the entire length of the rat small intestine. The receptor has been previously localized along the entire length of the mammalian gastrointestinal tract (Gama et al., 1997; Mitsuma et al., 1999; Cheng et al., 2002; Dufner et al., 2005; Kirchhoff and Geibel, 2006; Remy et al., 2007; Geibel, 2010), however, very few functional studies have been conducted on its role in fluid and electrolyte homeostasis in the small intestine. Using an *ex vivo* perfusion system that maintains the intestinal regions at 37°C throughout the experiment with a peristaltic flow, we can measure intestinal fluid secretion and absorption in real-time. As previously described, an increase in the fluorescence signal of FITC-inulin in the intraluminal perfusate corresponds to a decreased intraluminal fluid volume, which translates to absorption. In turn, a decrease in the fluorescence signal of FITC-inulin in the intraluminal perfusate responds to an increase in the intraluminal fluid. FITC-inulin fluorescence over time is thus indicative of intestinal luminal absorption or secretion (Munoz-Abraham et al., 2015).

The data obtained in the present study suggests that elevations in intraluminal Ca^2+^concentration can lead to a decrease in secretagogue induced fluid secretion and that further elevations in luminal Ca^2+^ concentration are associated with net fluid absorption via modulation in the CaSR located on the apical surface of the intestine. We confirm this hypothesis by application of a calcimimetic (AC-265347) with physiological Ca^2+^ concentration that results in decreased secretagogue induced fluid secretion comparable to a high Ca^2+^ perfusate. The observed effect of activating the CaSR established a dose-dependence and occurs along the entire length of the small intestine, with net fluid absorption occurring at a Ca^2+^ concentration of 7 mM in all intestinal regions (Figure 1). Accordingly, increasing the concentration of receptor ligand (Ca^2+^) increases the activity of the CaSR in modulating luminal fluid secretion, and at elevated levels of Ca^2+^ (7 mM) induces absorption of fluid.

Overstimulation of the secretory capacity of the intestinal tract results in secretory diarrhea, which is characterized by large stool volumes and is a leading cause of mortality amongst children and the elderly worldwide (Field, 2003; Schiller, 2009; Schiller et al., 2017). As such, there has been a search for therapies with the potential of directly inhibiting intestinal secretory mechanisms (Farthing, 2006). We thus sought to examine if activation of the CaSR by luminal Ca^2+^ would influence secretagogue-induced fluid secretion. FSK is a cyclic nucleotide activator that leads to an elevation of cAMP levels within intestinal epithelial cells and as such mimics the physiology of secretory diarrhea (Tang et al., 2016). As shown in Figure 2, increasing luminal Ca^2+^ concentration in the continued presence of FSK significantly decreases the secretory action of FSK in a dose-dependent manner. CaSR activation inhibits cyclic nucleotide activation and increases Na^+^-dependent absorption, thus inhibiting secretion and promoting fluid absorption. At a Ca^2+^ concentration of 7 mM, CaSR activation completely abolished FSK-induced fluid secretion along the small intestine. To confirm that the exposure to elevated luminal Ca^2+^ levels was via activation of the CaSR and not due to a non-targeted effect we exposed small intestinal segments to a physiological luminal Ca^2+^ concentration in the presence of the calcimimetic AC-265347 (Ma et al., 2011) (Figure 3). In the presence of FSK, intraluminal Ca^2+^ is just as effective as intraluminal AC-265347 in decreasing secretagogue-induced fluid secretion (Table 3 and Figure 3). In fact, 7 mM Ca^2+^ is significantly better than 2 μM AC-265347 in abrogating fluid secretion (Figure 4). These results confirm CaSR activation and are similar to what has been observed in the colon (Cheng, 2012; Tang et al., 2018).

The CaSR has recently provided a target for treatment of diarrheal illnesses, with the aim of activating it to induce its anti-secretory, pro-absorptive, anti-motility, and anti-inflammatory effects (Cheng, 2016). Human studies have shown that dietary Ca^2+^ improves resistance to enterotoxigenic *Escherichia coli* in young adults and ameliorates infectious diarrhea in immunocompromised children (Bovee-Oudenhoven et al., 2003; Cheng et al., 2013). Our findings corroborate these studies by providing direct evidence that the CaSR is functionally active along the entire length of the small intestine and acts as a modulator for fluid and electrolyte homeostasis. Additionally, we show that activation of the CaSR by Ca^2+^ leads to cessation of cyclic nucleotide-induced hypersecretion, and results in fluid absorption at higher luminal Ca^2+^ concentrations. These results not only provide a novel approach of Ca^2+^-based anti-secretory therapeutics, but also provides the possibility of correction of dehydration and nutrient absorption by increasing fluid absorption in secretory diarrheal maladies.

## Author Contributions

MB was involved in conducting the experiments, statistical analysis, and writing of the manuscript. RM was involved in conducting experiments, writing, and editing of the manuscript. TL and MF were involved in conducting the experiments and writing the manuscript. GC was involved in the experimental design. VB was involved in editing of the manuscript. FD and DM were involved in experimental design, manuscript writing, and editing. JG was involved in all aspects of the experimental design, statistical analysis, manuscript writing, and editing.

## Conflict of Interest Statement

The authors declare that the research was conducted in the absence of any commercial or financial relationships that could be construed as a potential conflict of interest.

## Abbreviations

AC-265347: 1-Benzothiazol-2-yl-1-(2,4-dimethyl-phenyl)-ethanol
CaSR: calcium-sensing receptor
FITC: fluorescein isothiocyanate
FSK: forskolin
GPCR: G-protein coupled receptor.
